# A rational cubic trigonometric approximation scheme of the generalized Cornu spirals

**DOI:** 10.1186/s40064-016-2544-3

**Published:** 2016-06-30

**Authors:** Hira Mahmood, Maria Hussain, Malik Zawwar Hussain

**Affiliations:** Department of Mathematics, University of the Punjab, Lahore, Pakistan; Lahore College for Women University, Lahore, Pakistan

**Keywords:** *G*^2^-approximation, *G*^1^-approximation, Generalized Cornu spirals, Rational cubic trigonometric Bézier curve, Relative curvature error, 68U05, 65D05, 65D07, 65D18

## Abstract

The $G^{2}$ and $G^{1}$-approximation schemes are introduced to approximate the popular generalized Cornu spirals with the help of the parametric rational cubic trigonometric Bézier curves. The $G^{2}$-approximation scheme has two free parameters whereas $G^{1}$-approximation scheme has four free parameters. To approximate the generalized Cornu spirals, the values of these free parameters are optimized by the minimization of the maximum relative curvature error of approximation. By comparing the relative curvature errors of approximation schemes, the developed approximation schemes are found less erroneous and more efficient than the existing GCS approximation schemes.

## Background

Monotonicity of curvature is a desirable feature in numerical curve and surface drawing applications e.g. in designing of robotic trajectories (Yang and Choi 2013), roads (Habib and Sakai 2007) and car bodies (Simon and Isik 1991). The generalized Cornu spirals (Cripps et al. 2010) are a family of spirals that have monotonic rational linear curvature profile. However their implementation in CAD systems is not possible due to their representation. The CAD system is based on simple polynomial curves, the integral and rational Bézier curves and B-spline curves. These curves are recursively defined and mimic the shape of control polygon so they are well suited for CAD. The only drawback is that the curvatures of Bézier and B-spline curves are not monotonic for randomly selected values of control points and weight functions. In this research paper, this void has been filled by constructing a parametric rational cubic trigonometric Bézier curve (RCTBC) approximation of the generalized Cornu spirals such that it has a monotonic curvature profile within a specified tolerance.

In the past few decades, efforts have been invested for approximating the special cases of GCS: Cornu spirals, logarithmic spirals and circular arcs. The curvature of Cornu spirals and logarithmic spirals also varies monotonically with respect to arc length. The parametric equations of the Cornu spirals are defined in terms of Fresnel integrals. Existing approximation schemes of Cornu spirals focus on the approximation of the Fresnel integrals, see Heald (1985) and Wang et al. (2001). Heald (1985) approximated the Fresnel integrals by rational polynomial function together with sine and cosine functions. The error function was the diagonal distance between the approximated and exact data points. Although, the approximation scheme of Cornu spirals proposed in Heald (1985) gave favourable results but the presence of arc length parameter made it unsuitable for CAD. Wang et al. (2001) approximated the Fresnel integral by Bernstein-Bézier polynomials of degree $N.$ The absolute error of approximation was computed by the Hausdorff distance between the exact and approximated points. In Wang et al. (2001), for favourable approximation the Bernstein–Bézier curve of at least degree seven was required. Baumgarm and Farin (1997) approximated the logarithmic spiral by rational cubic Bézier curves. The quality of approximation was considered by computing relative error between the total arc length of logarithmic spiral and rational cubic Bézier curve approximating it. In Fang (1998), Goldapp (1991) and Lee et al. (1996), the approximation of the unit quarter circle was carried out by minimizing the radius error of approximation. Lee et al. (1996) proposed $G^{0}$-approximation by quadratic Bézier curves, Goldapp (1991) proposed $G^{1}$-approximation by cubic Bézier curves and Fang (1998) proposed $G^{2}$-approximation by quintic Bézier curves. In Lee et al. (1996), Goldapp (1991) and Fang (1998), the radius error of approximation progressively decreased from $10^{ - 2}$ to $10^{ - 5}$ as the degree of the Bézier curve increased. Piegal and Tiller (2003) presented a B-spline approximation method for circular arcs. Since all the above mentioned approximation schemes focused on minimizing the Euclidean distance between the exact and approximated values so these fall short of controlling the curvature profile of the approximating curves.

The GCS (Cripps et al. 2010) is a continuous and smooth curve. It has a monotonic curvature and possesses only one inflection point at the most. These are the qualities which make it beneficial in the CAD system. GCS reduces to straight lines, circular arcs, logarithmic spirals or Cornu spirals for different values of parameters.

Cripps et al. (2010) proposed a $G^{2}$-approximation scheme for GCS. In Cripps et al. (2010), the authors formulated the approximating quintic Bézier curve as a function of four free parameters. The optimized values of these free parameters were computed by minimizing the maximum value of relative curvature error of approximation through a search routine. Thus the approximation scheme in Cripps et al. (2010) had hold over the curvature of the approximating curve. Cross and Cripps (2012) introduced a $G^{3}$-approximation scheme for generalized Cornu spirals using parametric quintic Bézier curves. Thus the $G^{3}$-approximation scheme (Cross and Cripps 2012) had only two degrees of freedom ($\beta_{1} ,\gamma_{1}$). The initial approximation of the shape factors $\beta_{1}$ and $\gamma_{1}$ were obtained by solving the nonlinear equations. If the initial approximation was unacceptable then a numerical search algorithm was applied repeatedly to obtain a reasonable value of the relative curvature error of approximation. Yoshida and Saito (2007) approximated the Quasi-Aesthetic Curves by parametric rational cubic Bézier curves. The Aesthetic Curves (Yoshida and Saito 2007) have monotone curvature profile. The values of free parameters were determined by minimizing the sum of the Euclidean distances between the points on rational cubic Bézier curve segments and the aesthetic curves.

Han et al. (2009) proposed the Bernstein–Bézier form of cubic trigonometric curves. The trigonometric curves developed in Han et al. (2009, 2010) were closer to the control polygon than the integral parametric cubic Bézier curves. Moreover, the review of literature (Han et al. 2009, 2010; Simon and Isik 1991), conveys that Bézier trigonometric curves are more efficient than the algebraic splines and ordinary parametric Bézier curves. Hussain et al. (2014a, b) used $GC^{1}$ trigonometric interpolants to preserve the shape of curve and surface data respectively.

In this research paper, $$ G^{k} ,k = 1,2, $$ approximation schemes are developed to approximate GCSs. The approximation is undertaken by the rational cubic trigonometric Bézier curve (RCTBC). The RCTBC was introduced in Hussain et al. (2015). The RCTBC enjoys all the properties of parametric rational cubic Bézier curve like convex-hull property, end point interpolation property, end tangent interpolation property. The quality of approximation by the RCTBC is better than ordinary rational and integral parametric cubic Bézier curves (Hussain et al. 2015). It is more flexible than non-rational ordinary parametric Bézier curves due to the presence of weight functions (Sederberg 2014). The presence of weight functions permits shape control. Apparently the trigonometric functions evaluations and presence of fraction in RCTBC looks like its demerits. It may be assumed that these functions will significantly increase the computation time of the developed $$ G^{2} $$ and $$ G^{1} $$ approximation schemes. But it is clear from Table 1, that the CPU time consumed by the developed $$ G^{2} $$ and $$ G^{1} $$ approximation schemes is less than 50 s. Moreover, softwares are available for the fast computation of trigonometric functions (Simon and Isik 1991). These characteristics of RCTBC (1) are the pushing force behind its use in this research paper for the approximation of GCS.

**Table 1:** Relative curvature errors and CPU time consumption

Approximation schemes	Maximum relative curvature error (*σ*)/CPU time (s)	Circular arc	Cornu spiral	Logarithmic spiral	Non-inflecting GCS	Normalized GCS
*G* ^2^-approximation (Theorem 1)	*σ*	9.7977 × 10^−8^	5.911 × 10^−4^	7.8252 × 10^−4^	5.52 × 10^−4^	0.0012
	CPU time	21.6380	17.769	39.0940	14.9760	21.1380
*G* ^1^-approximation (Theorem 2)	*σ*	5.62 × 10^−5^	8.94 × 10^−4^	3.60 × 10^−3^	4.10 × 10^−3^	0.0010
	CPU time	34.0870	20.230	45.0670	25.9800	26.9880

In this research paper, $$ G^{k} ,k = 1,2, $$ approximations of GCSs by the rational cubic trigonometric Bézier curve (1) are carried out in the following sequence:

- The developed $G^{1}$-approximation scheme evaluates all the control points of the RCTBC by matching the end points and end unit tangents of the GCS and RCTBC. The four weight functions and the distances ($d_{1} ,d_{3}$) are available as free parameters. Here $d_{1}$ is the distance between the first two control points of RCTBC, whereas, $d_{3}$ is the distance between its last two control points. Since the degrees of freedom of a Bézier curve is two less than its number of free parameters (Yoshida and Saito 2007). Therefore, two of the weight functions are fixed. The RCTBC is reformulated in terms of these newly computed values of control points and four free parameters.
- In $G^{2}$-approximation scheme, in addition to the control points, two out of total four weight functions are fixed by matching end points, end unit tangents and end curvatures of the GCS and the RCTBC. The remaining two weight functions and the distances between control points ($d_{1} ,d_{3}$) are the free parameters. Here again using the same arguments the weight functions are fixed. Thus the developed $G^{2}$-approximation scheme has two free parameters ($d_{1} ,d_{3}$). The order of continuity of $G^{1}$-approximation scheme is less than $G^{2}$-approximation scheme. But $G^{1}$-approximation scheme has more flexibility due to two more free parameters than $G^{2}$-approximation scheme.
- Since we are interested in developing a RCTBC with monotone curvature profile. Therefore, to control the curvature profile of the RCTBC, the relative curvature error is taken as the gauge for the optimal approximation. The optimal values of the free parameters of the developed $G^{2}$ and $G^{1}$-approximation schemes of GCS are obtained by minimizing the maximum value of the relative curvature error of these approximation schemes. This minimization is carried out by using the optimization tool box of MATLAB software based on sequential quadratic programming technique.
- The GCS is defined in terms of arc length parameter $s$ and RCTBC is a parametric curve of parameter $t \in \left[ {0,\frac{\pi }{4}} \right]$. In order to compare these curvatures both the curvatures should be reparametrized in terms of same parameter. Here, Algorithm 1 is proposed in “Re-parameterization of the GCS and the rational cubic trigonometric Bézier curve” section to find the points of the RCTBC corresponding to the points of the GCS. For comparison, the curvatures of RCTBC and GCS are evaluated simultaneously.
- The tolerances for the relative curvature error of the developed approximation schemes of GCS are determined by using non-rational cubic trigonometric Bézier curve. It is the least degree trigonometric Bézier curve which is completely evaluated by the $G^{2}$-approximation scheme without leaving any degree of freedom.
- The $G^{k}$-approximation schemes of GCS developed in this research paper are applied to the special cases of GCS (circular arc, Cornu spiral, logarithmic spiral, non-inflecting GCS, normalized GCS). The numerical results of approximations corresponding to these data sets are given in Table 1.

The affine transformations, rotation and translation, do not alter the curvature profile of the GCS. Therefore in this research paper, the $G^{k}$-approximation schemes are developed for the standard form of the GCS i.e. the initial point of the GCS is at origin $O\left( {0,0} \right)$ and the tangent at the initial point is in the direction of positive x-axis ($\theta \left( 0 \right) = 0$). It can be easily achieved for any given segment of GCS by first translating its initial point to origin then rotating it in the direction of positive x-axis.

By comparing the results in Table 1, it is concluded that the developed $G^{2}$ and $G^{1}$ approximation schemes of this research paper perform better than the prevailing approximation schemes of GCS (Cripps et al. 2010; Cross and Cripps 2012). Since the CPU time consumed by the developed approximation schemes is less than 50 s so it also rules out the assumption that the use of rational trigonometric Bézier curve destroys the efficiency of the developed $G^{2}$ and $G^{1}$ approximation schemes by greatly increasing the computation time.

## Preliminaries

In this section, the terms to be used in the rest of the paper are defined.

(i) Rational cubic trigonometric Bézier curves (RCTBC)

The rational cubic trigonometric Bézier curve (RCTBC) (Hussain et al. 2015) is a trigonometric alternative of well-known parametric rational cubic Bézier curve (Hoschek et al. 1993). The RCTBC is given by

1 $$ p\left( t \right) = \frac{{\mathop \sum \nolimits_{i = 0}^{3} b_{i}^{3} \left( t \right)\mu_{i} p_{i} }}{{\mathop \sum \nolimits_{i = 0}^{3} b_{i}^{3} \left( t \right)\mu_{i} }},\quad t \in \left[ {0,\frac{\pi }{4}} \right],\quad p_{i} \in {\mathbb{R}}^{2} . $$

Here $p_{i}$’s, $\mu_{i}$’s and $\frac{{b_{i}^{3} \left( t \right)\mu_{i} }}{{\mathop \sum \nolimits_{i = 0}^{3} b_{i}^{3} \left( t \right)\mu_{i} }},i = 0,1,2,3,$ are the control points, weight functions and the rational cubic trigonometric basis functions, respectively. The weight functions $\mu_{i}$ may have any real positive value. The cubic trigonometric functions $b_{i}^{3} \left( t \right),$ are given by

$$ b_{i}^{3} \left( t \right) = \left( {\begin{array}{*{20}c} 3 \\ i \\ \end{array} } \right)\left( {1 - tant} \right)^{3 - i} tan^{i}  t,\quad i = 0, 1, 2, 3 $$

It is clear from above definition that RCTBC (1) is a parametric curve. The RCTBC has the following properties:

- End point interpolation property: The RCTBC (1) interpolates the first and last control points i.e. $p\left( 0 \right) = p_{0}$ and $p\left( {\frac{\pi }{4}} \right) = p_{3} .$
- Convex-hull property: The sum of the rational cubic trigonometric basis functions $\frac{{b_{i}^{3} \left( t \right)\mu_{i} }}{{\mathop \sum \nolimits_{i = 0}^{3} b_{i}^{3} \left( t \right)\mu_{i} }},i = 0,1,2,3,$ is one and these are non-negative for $t \in \left[ {0,\frac{\pi }{4}} \right],\mu_{i} \in {\mathbb{R}}^{ + } .$ Therefore, the curve generated by RCTBC (1) always lies in the convex-hull of control points $p_{i} ,i = 0,1,2,3.$
- End tangents property: The first order derivatives of RCTBC (1) at the end points of the interval are given by
  $$ p'\left( 0 \right) = \frac{{3\mu_{1} \left( {p_{1} - p_{0} } \right)}}{{\mu_{0} }}\quad {\text{and}}\quad p'\left( {\frac{\pi }{4}} \right) = \frac{{6\mu_{2} \left( {p_{3} - p_{2} } \right)}}{{\mu_{3} }}. $$
- The RCTBC (1) is recursively defined and mimic the shape of control polygon.
- In Hussain et al. (2015), the quality of approximation of RCTBC (1) was measured and found better than the ordinary rational and integral parametric cubic Bézier curves.

The above highlighted properties of RCTBC (1) make it an ideal candidate for GCS approximation in CAD.

(ii) Non-rational cubic trigonometric Bézier curve

The parametric non-rational cubic trigonometric Bézier curve is defined as

2 $$ q\left( t \right) = \mathop \sum \limits_{i = 0}^{3} b_{i}^{3} \left( t \right)q_{i} ,\quad  t \in \left[ {0,\frac{\pi }{4}} \right],\quad q_{i} \in {\mathbb{R}}^{2} . $$

Here $q_{i}$ and $b_{i}^{3} \left( t \right)$ are the control points and trigonometric basis functions respectively. These cubic trigonometric basis functions,$b_{i}^{3} \left( t \right),$ are given by

$$ b_{i}^{3} \left( t \right) = \left( {\begin{array}{*{20}c} 3 \\ i \\ \end{array} } \right)\left( {1 - tant} \right)^{3 - i} tan^{i}  t,\quad i = 0,1,2,3. $$

It is a special case of (1) for $\mu_{i} = 1,i = 0,1,2,3.$

(iii) Curvature (Hoschek et al. 1993)

For a parametric curve $z\left( t \right) = \left( {x\left( t \right),y\left( t \right)} \right)$, the curvature $k_{z} \left( t \right)$ is given as follows

3 $$ k_{z} \left( t \right) = \frac{{\frac{dx}{dt}.\frac{{d^{2} y}}{dt} - \frac{dy}{dt}.\frac{{d^{2} x}}{dt}}}{{\left\{ {\left( {\frac{dx}{dt}} \right)^{2} + \left( {\frac{dy}{dt}} \right)^{2} } \right\}^{{\frac{3}{2}}} }} $$

(iv) $G^{1}$**-**approximation (Hoschek et al. 1993)

For the given parametric curves, $S_{1} \left( t \right)$ and $S_{2} \left( t \right)$, $S_{1} \left( t \right)$ is called $G^{1}$-approximation of $S_{2} \left( t \right)$ if both the curves have same end points and end unit tangents.

(v) $G^{2}$-approximation (Hoschek et al. 1993)

For the given parametric curves, $S_{1} \left( t \right)$ and $S_{2} \left( t \right)$, $S_{1} \left( t \right)$ is called $G^{2}$-approximation of $S_{2} \left( t \right)$ if both the curves have same end points, end unit tangents and end curvatures.

## Determining tolerance of relative curvature error

A GCS $r\left( s \right)$ is always bounded by two circular arcs, $c_{1} \left( s \right)$ and $c_{2} \left( s \right)$ (say). These circular arcs are completely defined by the two end points ($r\left( 0 \right)$ and $r\left( S \right)$) and the unit tangents at these end points ($t\left( 0 \right)$ and $t\left( S \right)$). Since the GCS has monotonic curvature profile so it is inside $c_{1} \left( s \right)$ and $c_{2} \left( s \right).$ In order to attain a good approximation of the GCS, the bounds of the relative curvature error of approximation need to be calculated. In this research paper, the approximation tolerance is established by approximating the two bounding circular arcs of GCS by non-rational cubic trigonometric Bézier curve (1). The non-rational curve (2) is the minimum degree trigonometric Bézier curve whose control points are completely determined by the $G^{2}$-approximation constraints. These non-rational cubic trigonometric Bézier approximations of circular arcs are used further for obtaining desired approximation of GCS.

Let the circular arcs have center at origin, the angle between the two radii is $\theta$ and the radius is $\rho .$ The $G^{2}$-approximation of the circular arc by the Bézier curve (2) is subject to the following conditions,

4 $$ q\left( 0 \right) = r\left( 0 \right),\quad  q\left( {\frac{\pi }{4}} \right) = r\left( S \right), $$

5 $$ \hat{T}\left( 0 \right) = t\left( 0 \right),\quad \hat{T}\left( {\frac{\pi }{4}} \right) = t\left( S \right), $$

6 $$ k_{q} \left( 0 \right) = k_{0} ,\quad k_{q} \left( {\frac{\pi }{4}} \right) = k_{1} . $$

**Fig. 1:**
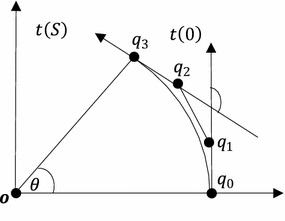
Approximation of circular arc by non-rational cubic trigonometric Bézier curve

Here $q\left( 0 \right)$ and $q\left( {\frac{\pi }{4}} \right)$ are the end points, $\hat{T}\left( 0 \right)$ and $\hat{T}\left( {\frac{\pi }{4}} \right)$ are the end unit tangents, $k_{q} \left( 0 \right)$ and $k_{  q} \left( {\frac{\pi }{4}} \right)$ are the end curvatures of the Bézier curve (2). The curvatures of the circular arc at the initial and final points of the domain are $k_{0}$ and $k_{1}$ respectively (Fig. 1).

Substituting the values of $r\left( 0 \right)$, $r\left( S \right),t\left( 0 \right)$, $t\left( S \right)$, $\hat{T}\left( 0 \right)$ and $\hat{T}\left( {\frac{\pi }{4}} \right)$ in (4) and (5), we get

7 $$ q_{0} = \left( {\rho ,0} \right),\quad q_{3} = \left( {\rho cos \theta ,\rho sin \theta } \right), $$

8 $$ \frac{{q_{1} - q_{0} }}{\|{q_{1} - q_{0} }\|} = \left( {0,1} \right),\quad    \frac{{q_{3} - q_{2} }}{\|{q_{3} - q_{2} }\|} = \left( { - sin \theta ,cos \theta } \right). $$

From (7) and (8), the control points of the cubic Bézier (2) are evaluated such that $q_{0} \left( {\rho ,0} \right)$, $q_{1} \left( {\rho ,\rho \alpha } \right)$, $q_{2} \left( {\rho \left( {cos \theta + sin \theta } \right), \rho \left( {sin \theta - \beta cos \theta } \right)} \right)$, $q_{3} \left( {\rho cos \theta ,\rho sin \theta } \right).$ Here, $\alpha = \frac{\|{q_{1} - q_{0} }\|}{\rho }$ and $\beta = \frac{\|{q_{3} - q_{2} }\|}{\rho }$ are the free parameters. By the choice of the free parameters, these are always positive.

The curvature of the Bézier curve (2), $k_{q} \left( t \right)$, as computed by the (3) is

9 $$ k_{q} \left( t \right) = \frac{f\left( t \right)}{\rho }. $$

Here,

$$ \begin{aligned} f\left( t \right) & = \frac{g\left( t \right)}{{\left\{ {\left( {h\left( t \right)} \right)^{2} + \left( {w\left( t \right)} \right)^{2} } \right\}^{{\frac{3}{2}}} }} , \\ g\left( t \right) & = \left( {1 - cos \theta - \beta sin \theta } \right)\left( {\left( {1 - tan t} \right)^{3} sec^{6} t \alpha } \right) + \left( {1 - cos \theta - \alpha sin \theta } \right) \\ & \quad \times \left( {tan^{3}t \,sec^{6} t \beta } \right) + \left( {1 - cos\theta } \right) \\ & \quad \times \left( {\left( {1 - tan t} \right)^{2} tan t \,sec^{6} t \alpha + \left( {1 - tan t} \right)tan^{2} t\,sec^{6} t \beta } \right), \\ h\left( t \right) & = \left( {2\left( {1 - tan t} \right)tan t \,sec^{2} t } \right)\left( {cos \theta + \beta sin \theta - 1} \right) - \beta  tan^{2} t \,sec^{2} t\,sin \theta ), \\ w\left( t \right) & = \left( {1 - tan t} \right)^{2} sec^{2} t \alpha + \left( {2\left( {1 - tan t} \right)tan t\,sec^{2} t } \right)\left( {sin \theta - \beta cos \theta - \alpha } \right) \\ & \quad + (\beta  tan^{2} t \,sec^{2} t\,cos \theta ). \\ \end{aligned} $$

Substituting the values of $k_{0}$ and $k_{1}$ in (6) leads to

10 $$ k_{q} \left( 0 \right) = \frac{1}{\rho },\quad k_{q} \left( {\frac{\pi }{4}} \right) = \frac{1}{\rho }, $$

where,

11 $$ k_{q} \left( 0 \right) = \frac{{2\left( {1 - cos \theta - \beta sin \theta } \right)}}{{3\alpha^{2} \rho }}, \quad k_{q} \left( {\frac{\pi }{4}} \right) = \frac{{2\left( {1 - cos \theta - \alpha sin \theta } \right)}}{{3\beta^{2} \rho }}. $$

Substituting the values of end curvatures from (11) in (10), the following set of equations are achieved,

12 $$ 2\left( {1 - cos \theta } \right) = 3\alpha^{2} + 2\beta sin \theta , $$

13 $$ 2\left( {1 - cos \theta } \right) = 3\beta^{2} + 2\alpha sin \theta . $$

It results in

$$ \left( {\alpha - \beta } \right)\left( {\alpha + \beta - \frac{2}{3}sin \theta } \right) = 0. $$

Now, either $\alpha = \beta$ or $\alpha + \beta = \frac{2}{3}sin \theta .$

### **Case 1**

$$ \alpha = \beta $$.

If $\alpha = \beta ,$ then (12) can be written as

14 $$ 3\alpha^{2} + 2\alpha sin \theta - 2\left( {1 - cos \theta } \right) = 0. $$

Solving the quadratic Eq. (14), the two roots $\alpha_{1 }$ and $\alpha_{2}$ are as follows,

$$ \alpha_{1} = \frac{{2sin \frac{\theta }{2}\left( { - cos \frac{\theta }{2} + \sqrt {3 + cos^{2} \frac{\theta }{2}} } \right)}}{3},\quad \alpha_{2} = \frac{{2sin \frac{\theta }{2}\left( { - cos \frac{\theta }{2} - \sqrt {3 + cos^{2} \frac{\theta }{2}} } \right)}}{3}. $$

It can be easily seen that for $0 < \theta \le \frac{\pi }{2}, \alpha_{2} < 0$, a contradiction to the choice of α’s. Hence $\alpha_{1}$ is the only root of (14). The only solution of the simultaneous Eqs. (12) and (13) is ($\alpha_{1} , \beta_{1}$), where $\alpha_{1} = \beta_{1} .$

### **Case 2**

$$ \alpha + \beta = \frac{2}{3}sin \theta $$

If $\alpha + \beta = \frac{2}{3}sin \theta$, then (12) can be written as,

$$ 3\alpha^{2} + 2\beta sin \theta - 2\left( {1 - cos \theta } \right) = 0. $$

In this case, simultaneous Eqs. (12) and (13) have the solution set: $\left\{ {\left( {\alpha_{3} ,\beta_{3} } \right),\left( {\alpha_{4} ,\beta_{4} } \right)} \right\}$, where

$$ \begin{aligned} \alpha_{3} & = \frac{{2sin \frac{\theta }{2}\left( {cos \frac{\theta }{2} - \sqrt 3 sin \frac{\theta }{2}} \right)}}{3},\quad \beta_{3} = \frac{{2sin \frac{\theta }{2}\left( {cos \frac{\theta }{2} + \sqrt 3 sin \frac{\theta }{2}} \right)}}{3}, \\ \alpha_{4} & = \frac{{2sin \frac{\theta }{2}\left( {cos \frac{\theta }{2} + \sqrt 3 sin \frac{\theta }{2}} \right)}}{3},\quad \beta_{4} = \frac{{2sin \frac{\theta }{2}\left( {cos \frac{\theta }{2} - \sqrt 3 sin \frac{\theta }{2}} \right)}}{3}. \\ \end{aligned} $$

It can be easily verified that $cos \frac{\theta }{2} > \sqrt 3 sin \frac{\theta }{2}$ for $0 < \theta \le \frac{\pi }{3}$ and $cos \frac{\theta }{2} < \sqrt 3 sin \frac{\theta }{2}$ for $\frac{\pi }{3} < \theta \le \frac{\pi }{2}.$ Since $\alpha ,\beta > 0$ and $0 < \theta \le \frac{\pi }{2}$ so if $\frac{\pi }{3} < \theta \le \frac{\pi }{2},$ the only solution is $\left( {\alpha_{1} ,\beta_{1} } \right)$ given in Case 1. However, for $0 < \theta \le \frac{\pi }{3}$, there are three solutions of (12) and (13) i.e. $\left( {\alpha_{1} ,\beta_{1} } \right), \left( {\alpha_{3} ,\beta_{3} } \right)$ and $\left( {\alpha_{4} ,\beta_{4} } \right)$. Thus we have three distinct non-rational cubic trigonometric Bézier approximations (2) of the circular arcs. Now to establish the acceptable tolerance of the relative curvature error of approximation, the relative curvature error of approximation of circular arc is given by

15 $$ \delta \left( t \right) = \frac{{\left| {k_{q} \left( t \right) - \frac{1}{\rho }} \right|}}{{max\left\{ {\left| {k_{q} \left( t \right)} \right|,\left| {\frac{1}{\rho }} \right|} \right\}}}. $$

From (9), (15) is rewritten as

16 $$ \delta \left( t \right) = \frac{{\left| {f\left( t \right) - 1} \right|}}{{max\left\{ {\left| {f\left( t \right)} \right|,1} \right\}}}. $$

The three maximum relative curvature errors of approximation corresponding to three non-rational cubic trigonometric Bézier approximating curves of circular arc are given by

17 $$ \varepsilon_{i} = max\left\{ {\delta_{i} \left( t \right)} \right\},\quad t \in \left[ {0,\frac{\pi }{4}} \right],\quad i = 1,2,3. $$

The smallest tolerance is $\bar{\varepsilon } = min\left\{ {\varepsilon_{i} } \right\},i = 1,2,3.$ However, by performing the results it is observed that $\bar{\varepsilon }$ is obtained when $\alpha = \beta .$ Thus corresponding to the two non-rational cubic trigonometric Bézier approximations of the bounding circular arcs, two curvature tolerances $\bar{\varepsilon }_{1}$ and $\bar{\varepsilon }_{2}$ are obtained. However the practical acceptable relative curvature error tolerance $\varepsilon$ is given by

18 $$ \varepsilon = 2max\left\{ {\bar{\varepsilon }_{1} ,\bar{\varepsilon }_{2} } \right\}. $$

In Cripps et al. (2010), the tolerance of relative curvature error less than $0.05$ was considered acceptable.

## $G^{k}$**-**approximation of GCS by rational cubic trigonometric Bézier curve

In the following section, the proposed $G^{2}$ and $G^{1}$-approximation schemes of GCS by RCTBC (1) are presented.

### $G^{2}$-approximation of GCS

The $G^{2}$-approximation of GCS is carried out by the following set of equations,

19 $$ p\left( 0 \right) = \tilde{r}\left( 0 \right), \quad  p\left( {\frac{\pi }{4}} \right) = \tilde{r}\left( S \right), $$

20 $$ \tilde{T}\left( 0 \right) = \tilde{t}\left( 0 \right), \quad \tilde{T}\left( {\frac{\pi }{4}} \right) = \tilde{t}\left( S \right), $$

21 $$ k_{p} \left( 0 \right) = \tilde{k}_{0} ,\quad  k_{p} \left( {\frac{\pi }{4}} \right) = \tilde{k}_{1} . $$

Here $p\left( 0 \right)$ and $p\left( {\frac{\pi }{4}} \right)$ are the end points, $\tilde{T}\left( 0 \right)$ and $\tilde{T}\left( {\frac{\pi }{4}} \right)$ are the end unit tangents, $k_{p} \left( 0 \right)$ and $k_{p} \left( {\frac{\pi }{4}} \right)$ are the end curvatures of the RCTBC (1). The curvature of the GCS at the initial and final points of the domain are $\tilde{k}_{0}$ and $\tilde{k}_{1}$ respectively. The GCS is defined by the two end points, $\tilde{r}\left( 0 \right) = \left( {0,0} \right)$ and $\tilde{r}\left( S \right) = \left( {x\left( S \right),y\left( S \right)} \right)$, and the unit tangents at these end points are $\tilde{t}\left( 0 \right) = \left( {1,0} \right)$ and $\tilde{t}\left( S \right) = \left( {{ cos }\theta \left( S \right),sin\theta \left( S \right)} \right)$. Here $\theta \left( S \right)$ is the angle made by $\tilde{t}\left( S \right)$ with x-axis. Substituting these values in (19), (20) and (21) we have

22 $$ p_{0} = \left( {x\left( 0 \right),y\left( 0 \right)} \right),\quad p_{3} = \left( {x\left( S \right),y\left( S \right)} \right), $$

23 $$ \frac{{p_{1} - p_{0} }}{\|{p_{1} - p_{0} }\|} = \tilde{t}\left( 0 \right),\quad \frac{{p_{3} - p_{2} }}{\|{p_{3} - p_{2} }\|} = \tilde{t}\left( S \right), $$

24 $$ \frac{{2\mu_{0} \mu_{2} \left( {y\left( S \right) - d_{3} sin \theta \left( S \right)} \right)}}{{3\mu_{1}^{2} d_{1}^{2} }} = \tilde{k}_{0} , \quad \frac{{2\mu_{1} \mu_{3} sin \theta \left( S \right)\left( {x\left( S \right) - d_{1} } \right) - y\left( S \right)cos \theta \left( S \right)}}{{3\mu_{2}^{2} d_{3}^{2} }} = \tilde{k}_{1.} $$

Here, $$ d_{i} = \|p_{i} - p_{i - 1}\| ,\left( {i = 1,2,3} \right), $$ and $$ \mu_{i} > 0 \left( {i = 0,1,2,3} \right) $$ are the weight functions of RCTBC (1). By solving (22), (23) and (24), the values of control points $$ (p_{i} , i = 0,1,2,3) $$ and weight functions $$ \mu_{0} $$, $$ \mu_{3} $$ of the RCTBC (1) in terms of $$ d_{1} $$, $$ d_{3} , $$$$ \mu_{1} $$ and $$ \mu_{2} $$ are obtained. The values of control points thus obtained are

25 $$ p_{0} = \left( {0,0} \right),\quad p_{1} = \left( {d_{1} , 0} \right),\quad p_{2} = \left( {x\left( S \right) - d_{3} cos \theta \left( S \right),y\left( S \right) - d_{3} sin \theta \left( S \right)} \right),\quad p_{3} = \left( {x\left( S \right),y\left( S \right)} \right). $$

The weights $\mu_{0}$ and $\mu_{3}$ are given by

26 $$ \mu_{0} = \frac{{3\mu_{1}^{2} d_{1}^{2} \tilde{k}_{0} }}{{2\mu_{2} \left( {y\left( S \right) - d_{3} sin \theta \left( S \right)} \right)}},\quad \mu_{3} = \frac{{3\mu_{2}^{2} d_{3}^{2} \tilde{k}_{1} + y\left( S \right)cos \theta \left( S \right)}}{{2\mu_{1} sin \theta \left( S \right)\left( {x\left( S \right) - d_{1} } \right)}}. $$

Here, $d_{1}$, $d_{3}$, $\mu_{1}$ and $\mu_{2}$ are the free parameters. The optimized values of these free parameters can be obtained by minimizing the maximum relative curvature error of $G^{2}$-approximation schemes via any optimization technique.

#### Minimization of the curvature

For the control points given in (25), the parametric equations of RCTBC (1) are

27 $$ x\left( t \right) = \frac{{x_{1} \left( t \right)}}{F\left( t \right)}\quad {\text{and}}\quad y\left( t \right) = \frac{{y_{1} \left( t \right)}}{F\left( t \right)}. $$

Here,

$$ \begin{aligned} x_{1} \left( t \right) & = 3\left( {1 - tan t} \right)^{2} tan t\, d_{1} \mu_{1} + 3\left( {1 - tan\,t} \right)tan^{2} t \left( {x\left( S \right) - d_{3} \,cos \theta \left( S \right)} \right)\mu_{2} + tan^{3} t x\left( S \right) \mu_{3} , \\ y_{1} \left( t \right) & = 3\left( {1 - tan t} \right)tan^{2} t \left( {y\left( S \right) - d_{3} sin \theta \left( S \right)} \right)\mu_{2} + tan^{3} t x\left( S \right) \mu_{3} , \\ F\left( t \right) & = \left( {1 - tan t} \right)^{3} \mu_{0} + 3\left( {1 - tan t} \right)^{2} tan t \,\mu_{1} + 3\left( {1 - tan t} \right)tan^{2} t \,\mu_{2} + tan^{3} t  \mu_{3} . \\ \end{aligned} $$

The first and second order derivatives of $x\left( t \right)$ and $y\left( t \right)$ are given by

28 $$ \frac{dx}{dt} = \frac{E\left( t \right)}{{F\left( t \right)^{2} }},\quad \frac{dy}{dt} = \frac{G\left( t \right)}{{F\left( t \right)^{2} }}, $$

29 $$ \frac{{d^{2} x}}{{dt^{2} }} = \frac{H\left( t \right)}{{F\left( t \right)^{3} }},\quad \frac{{d^{2} y}}{{dt^{2} }} = \frac{I\left( t \right)}{{F\left( t \right)^{3} }}. $$

Here,

$$ \begin{aligned} E\left( t \right) & = \left( {1 - tan t} \right)^{5} sec ^{2} t a_{0} + \left( {1 - tan t} \right)^{4} tan t\,sec ^{2} t a_{1} + \left( {1 - tan t} \right)^{3} \\ & \quad \times tan^{2}  t \,sec ^{2} t a_{2} + \left( {1 - tan t} \right)^{2}  tan ^{3} t \,sec ^{2} t a_{3} + \left( {1 - tan t} \right)tan ^{4} t \,sec ^{2} t a_{4} \\ & \quad + tan^{5}  t \,sec ^{2} t a_{5} , \\ G\left( t \right) & = \left( {1 - tan t} \right)^{5} sec ^{2} t b_{0} + \left( {1 - tan t} \right)^{4} tan t\, sec ^{2} t b_{1} + \left( {1 - tan t} \right)^{3} tan^{2}  t \\ & \quad \times sec ^{2} t b_{2} + \left( {1 - tan t} \right)^{2}  tan ^{3} t \,sec ^{2} t b_{3} + \left( {1 - tan t} \right)tan ^{4} t \,sec ^{2} t b_{4} \\ & \quad + tan^{5}  t \,sec ^{2} t b_{5} , \\ H\left( t \right) & = \left( {1 - tan t} \right)^{7} sec ^{4} t c_{0} + \left( {1 - tan t} \right)^{6} tan t\,sec ^{4} t c_{1} + \left( {1 - tan t} \right)^{8} tan t\,sec ^{2} t c_{2} \\ & \quad + \left( {1 - tan t} \right)^{7} tan^{2} t\,sec ^{2} t c_{3} + \left( {1 - tan t} \right)^{6} tan^{3} t\,sec ^{2} t c_{4} + \left( {1 - tan t} \right)^{5} \\ & \quad \times tan^{2} t\,sec ^{4} t c_{5} + \left( {1 - tan t} \right)^{5} tan^{4} t\,sec ^{2} t c_{6} + \left( {1 - tan t} \right)^{4} tan^{3} t\,sec ^{4} tc_{7} \\ & \quad + \left( {1 - tan t} \right)^{4} tan^{5} t\,sec ^{2} t c_{8} + \left( {1 - tan t} \right)^{3} tan^{4} t\,sec ^{4} t c_{9} + \left( {1 - tan t} \right)^{3} \\ & \quad \times tan^{6} t\,sec ^{2} t c_{10} + \left( {1 - tan t} \right)^{2} tan^{5} t\,sec ^{4} t c_{11} + \left( {1 - tan t} \right)^{2} tan^{7} t\,sec ^{2} t c_{12} \\ & \quad + \left( {1 - tan t} \right)tan^{6} t\,sec ^{4} t c_{13} + \left( {1 - tan t} \right)tan^{8} t\,sec ^{2} t c_{14} + tan^{9} t\,sec ^{2} t c_{15} \\ & \quad + tan^{7} t\,sec ^{4} t c_{16} , \\ I\left( t \right) & = \left( {1 - tan t} \right)^{7} sec ^{4} t e_{0} + \left( {1 - tan t} \right)^{6} tan t\,sec ^{4} t e_{1} + \left( {1 - tan t} \right)^{8} tan t\,sec ^{2} t e_{2} \\ & \quad + \left( {1 - tan t} \right)^{7} tan^{2} t\,sec ^{2} t e_{3} + \left( {1 - tan t} \right)^{6} tan^{3} t\,sec ^{2} t e_{4} + \left( {1 - tan t} \right)^{5} \\ & \quad \times tan^{2} t\,sec ^{4} t e_{5} + \left( {1 - tan t} \right)^{5} tan^{4} t\,sec ^{2} t e_{6} + \left( {1 - tan t} \right)^{4} tan^{3} t\,sec ^{4} t e_{7} \\ & \quad + \left( {1 - tan t} \right)^{4} tan^{5} t\,sec ^{2} t e_{8} + \left( {1 - tan t} \right)^{3} tan^{4} t\,sec ^{4} t e_{9} + \left( {1 - tan t} \right)^{3} \\ & \quad \times tan^{6} t\,sec ^{2} t e_{10} + \left( {1 - tan t} \right)^{2} tan^{5} t\,sec ^{4} t e_{11} + \left( {1 - tan t} \right)^{2} tan^{7} t\,sec ^{2} t e_{12} \\ & \quad + \left( {1 - tan t} \right)tan^{6} t\,sec ^{4} t e_{13} + \left( {1 - tan t} \right)tan^{8} t\,sec ^{2} t e_{14} + tan^{9} t\,sec ^{2} t e_{15} \\ & \quad + tan^{7} t\,sec ^{4} t e_{16} , \\ \end{aligned} $$

$$ \begin{aligned} a_{0} & = 3\mu_{0} \mu_{1} d_{1} , \\ a_{1} & = 3\mu_{0} \mu_{1} d_{1} + 6\mu_{0} \mu_{2} \left( {x\left( S \right) - d_{3} cos \theta \left( s \right)} \right), \\ a_{2} & = - 9\mu_{1} \mu_{2} d_{1} + \left( {6\mu_{0} \mu_{2} + 9\mu_{1} \mu_{2} } \right)\left( {x\left( S \right) - d_{3} cos \theta \left( s \right)} \right) + 3\mu_{0} \mu_{3} x\left( S \right), \\ a_{3} & = 9\mu_{1} \mu_{2} \left( {x\left( S \right) - d_{3} cos \theta \left( s \right)} \right)  - \left( {9\mu _{1} \mu _{2}  + 6\mu _{1} \mu _{3} } \right)d_{1}  + (3\mu _{0} \mu _{3}  + 6\mu _{1} \mu _{3} )x\left( S \right), \\ a_{4} & = - 6\mu_{1} \mu_{3} d_{1} - 3\mu_{2} \mu_{3} \left( {x\left( S \right) - d_{3} cos \theta \left( s \right)} \right) + \left( {6\mu_{1} \mu_{3} + 3\mu_{2} \mu_{3} } \right)x\left( S \right), \\ a_{5} & = 3\mu_{2} \mu_{3} d_{3} cos \theta \left( s \right), \\ b_{0} & = 0, \\ b_{1} & = 6\mu_{0} \mu_{2} \left( {y\left( S \right) - d_{3} sin \theta \left( s \right)} \right), \\ b_{2} & = \left( {6\mu_{0} \mu_{2} + 9\mu_{1} \mu_{2} } \right)\left( {y\left( S \right) - d_{3} sin \theta \left( s \right)} \right) + 3\mu_{0} \mu_{3} y\left( S \right), \\ b_{3} & = 9\mu_{1} \mu_{2} \left( {y\left( S \right) - d_{3} sin \theta \left( s \right)} \right) + \left( {3\mu_{0} \mu_{3} + 6\mu_{1} \mu_{3} } \right)y\left( S \right), \\ b_{4} & = - 3\mu_{2} \mu_{3} \left( {y\left( S \right) - d_{3} sin \theta \left( s \right)} \right) + \left( {6\mu_{1} \mu_{3} + 3\mu_{2} \mu_{3} } \right)y\left( S \right), \\ b_{5} & = 3\mu_{2} \mu_{3} d_{3} sin \theta \left( s \right), \\ \end{aligned} $$

$$ \begin{aligned} c_{0} & = \left( {18\mu_{0} \mu_{1} \left( {\mu_{0} - \mu_{1} } \right) - 12\mu_{0}^{2} \mu_{1} } \right)d_{1} + 6\mu_{0}^{2} \mu_{2} \left( {x\left( S \right) - d_{3} cos \theta \left( S \right)} \right), \\ c_{1} & = 6\mu_{0}^{2} \mu_{3} x\left( S \right) + 24\mu_{0}^{2} \mu_{2} \left( {x\left( S \right) - d_{3} cos \theta \left( S \right)} \right) - 6\mu_{0} \left( {9\mu_{1} \mu_{2} - \mu_{0} \mu_{1} + 3\mu_{0}^{2} } \right)d_{1} , \\ c_{2} & = 6\mu_{0}^{2} \mu_{1} d_{1} , \\ c_{3} & = \left( {6\mu_{0} \left( {3\mu_{1}^{2} + \mu_{0} \mu_{1} } \right)} \right)d_{1} + 12\mu_{0}^{2} \mu_{2} (x\left( S \right) - d_{3} cos \theta \left( S \right), \\ c_{4} & = 6\mu_{0}^{2} \mu_{3} x\left( S \right) + 18\mu_{1}^{2} \mu_{0} d_{1} + \left( {12\mu_{0}^{2} \mu_{2} + 9\mu_{1} \mu_{2} } \right)\left( {x\left( S \right) - d_{3} cos \theta \left( S \right)} \right), \\ c_{5} & = 18\mu_{0} \left( {\mu_{0} \mu_{3} + \mu_{1} \mu_{3} } \right)x\left( S \right) + 6\mu_{2} \left( {6\mu_{0}^{2} - \mu_{2} + \mu_{1} } \right)\left( {x\left( S \right) - d_{3} cos \theta \left( S \right)} \right) - 36\mu_{0} \mu_{1} \\ & \quad \times \left( {3\mu_{2} + \mu_{3} } \right)d_{1} , \\ c_{6} & = 3\mu_{0} \mu_{3} \left( {2\mu_{0} + 10\mu_{1} } \right)x\left( S \right) + 36\mu_{2} \left( {\mu_{0} \mu_{2} + 3\mu_{1}^{2} + 3\mu_{0} \mu_{1} } \right)(x\left( S \right) - d_{3} cos \theta \left( S \right) - 6\mu_{1} \\ & \quad \times \left( {9\mu_{1} \mu_{2} + \mu_{0} \mu_{3} } \right)d_{1} , \\ c_{7} & = 2\mu_{3} \left( {6\mu_{0}^{2} + 33\mu_{0} \mu_{1} - 3\mu_{0} \mu_{2} + 9\mu_{1}^{2} } \right)x\left( s \right) \\ & \quad + 2\mu_{2} \left( {27\mu_{1}^{2} - 27\mu_{1} \mu_{2} - 36\mu_{0} \mu_{2} + 27\mu_{0} \mu_{1} - 21\mu_{0} \mu_{3} } \right)\left( {x\left( S \right) - d_{3} cos \theta \left( S \right)} \right) \\ & \quad + 2u1\left( { - 27\mu_{1} \mu_{2} - 9\mu_{1} \mu_{3} + 27\mu_{2}^{2} - 27\mu_{0} \mu_{2} - 39\mu_{0} \mu_{3} } \right)d_{1} , \\ c_{8} & = 6\mu_{3} \left( {6\mu_{1}^{2} + 5\mu_{0} \mu_{1} + 4\mu_{0} \mu_{2} } \right)x\left( S \right) \\ & \quad + 6\mu_{2} \left( {6\mu_{0} \mu_{2} + 9\mu_{1} \mu_{2} + 9\mu_{1}^{2} + \mu_{0} \mu_{3} } \right)\left( {x\left( S \right) - d_{3} cos \theta \left( S \right)} \right) \\ & \quad + 6\mu_{1} \left( { - 9\mu_{1} \mu_{2} - 6\mu_{3} u1 - 9\mu_{2}^{2} - \mu_{0} \mu_{3} } \right)d_{1} ,  \end{aligned} $$

$$\begin{aligned} c_{9} & = 12\mu_{3} \left( {6\mu_{1}^{2} + 4\mu_{0} \mu_{1} - \mu_{0} \mu_{3} + \mu_{0} \mu_{2} } \right)x\left( S \right) \\ & \quad + 6\mu_{2} \left( { 9\mu_{1}^{2} - 9\mu_{1} \mu_{2} - 9\mu_{0} \mu_{2} - 13\mu_{0} \mu_{3} - 9\mu_{1} \mu_{3} } \right)\left( {x\left( S \right) - d_{3} cos \theta \left( S \right)} \right) \\ & \quad + 6 \mu_{1} \left( { - 9\mu_{1} \mu_{2} - 12\mu_{1} \mu_{3} + 9\mu_{2}^{2} + 9\mu_{2} \mu_{3} - 7\mu_{0} \mu_{3} } \right)d_{1} , \\ c_{10} & = 6\mu_{3} \left( {6\mu_{1}^{2} + 9\mu_{1} \mu_{2} + \mu_{0} \mu_{3} + 4\mu_{0} \mu_{2} } \right)x\left( S \right) + 6\mu_{2} \left( {9\mu_{1} \mu_{2} + \mu_{0} \mu_{3} } \right)\left( {x\left( S \right) - d_{3} cos \theta \left( S \right)} \right) \\ & \quad + 6\left( { - 6\mu_{1} \mu_{3} - 9u2^{2} - 9u3u2} \right)d_{1} , \\ c_{11} & = 18\mu_{3} \left( {\mu_{0} \mu_{2} - \mu_{0} \mu_{3} + 3\mu_{1} \mu_{2} - \mu_{1} \mu_{3} + 3\mu_{1}^{2} } \right)x\left( S \right) + 18\mu_{3} \left( {3\mu_{1} \mu_{2} + \mu_{1} \mu_{3} - 3\mu_{1}^{2} } \right)d_{1} \\ & \quad + 6\mu_{2} \left( { - 6\mu_{0} \mu_{3} + 6\mu_{1} } \right)\left( {x\left( S \right) - d_{3} cos \theta \left( S \right)} \right), \\ c_{12} & = 6\mu_{3} \left( {3\mu_{2}^{2} + 9\mu_{1} \mu_{2} + \mu_{0} \mu_{3} + 2\mu_{1} \mu_{3} } \right)x\left( S \right) - 6\mu_{3} \left( {9\mu_{1} \mu_{2} + 2\mu_{1} \mu_{3} } \right)d_{1} \\ & \quad - 18\mu_{2}^{2} \mu_{3} \left( {x\left( S \right) - d_{3} cos \theta \left( S \right)} \right), \\ c_{13} & = - 6\mu_{3} \left( {\mu_{0} \mu_{3} - 9\mu_{1} \mu_{2} + 4\mu_{1} \mu_{3} + \mu_{2} \mu_{3} - 3\mu_{2}^{2} } \right)x\left( S \right) + 24\mu_{1} \mu_{3}^{2} d_{1} + ( - 18\mu_{3} \mu_{2}^{2} \\ & \quad + 9\mu_{1} \mu_{2} - \mu_{2} \mu_{3} )\left( {x\left( S \right) - d_{3} cos \theta \left( S \right)} \right), \\ c_{14} & = 6\mu_{3} \left( {3\mu_{2}^{2} + \mu_{2} \mu_{3} + 2\mu_{1} \mu_{3} } \right)x\left( S \right) - 12\mu_{1} \mu_{3}^{2} d_{1} - 6\mu_{3} \left( {3\mu_{2}^{2} + \mu_{2} \mu_{3} } \right) \\ & \quad \times \left( {x\left( S \right) - d_{3} cos \theta \left( S \right)} \right) \\ c_{15} & = 6\mu_{2} \mu_{3}^{2} x\left( S \right) - 6\mu_{2} \mu_{3}^{2} \left( {x\left( S \right) - d_{3} cos \theta \left( S \right)} \right), \\ c_{16} & = - 6\mu_{3} \left( { - 3\mu_{2}^{2} + \mu_{2} \mu_{3} + \mu_{1} \mu_{3} } \right)x\left( S \right) + 6\mu_{1} \mu_{3}^{2} d_{1} - 6\mu_{3} \left( {3\mu_{2}^{2} - \mu_{2} \mu_{3} } \right) \\ & \quad \times \left( {x\left( S \right) - d_{3} cos \theta \left( S \right)} \right), \end{aligned}$$

$$ \begin{aligned} e_{0} & = 6\mu_{0}^{2} \mu_{2} \left( {y\left( S \right) - d_{3} sin \theta \left( S \right)} \right), \\ e_{1} & = 6\mu_{0}^{2} \mu_{3} y\left( S \right) + 24\mu_{0}^{2} \mu_{2} \left( {y\left( S \right) - d_{3} sin \theta \left( S \right)} \right), \\ e_{2} & = 0, \\ e_{3} & = 12\mu_{0}^{2} \mu_{2} (y\left( S \right) - d_{3} sin \theta \left( S \right), \\ e_{4} & = 6\mu_{0}^{2} \mu_{3} y\left( S \right) + \left( {12\mu_{0}^{2} \mu_{2} + 9\mu_{1} \mu_{2} } \right)\left( {y\left( S \right) - d_{3} sin \theta \left( S \right)} \right), \\ e_{5} & = 18\mu_{0} \left( {\mu_{0} \mu_{3} + \mu_{1} \mu_{3} } \right)y\left( S \right) + 6\mu_{2} \left( {6\mu_{0}^{2} - \mu_{2} + \mu_{1} } \right)\left( {y\left( S \right) - d_{3} sin \theta \left( S \right)} \right), \\ e_{6} & = 3\mu_{0} \mu_{3} \left( {2\mu_{0} + 10\mu_{1} } \right)y\left( S \right) + 36\mu_{2} \left( {\mu_{0} \mu_{2} + 3\mu_{1}^{2} + 3\mu_{0} \mu_{1} } \right)(y\left( S \right) - d_{3} sin \theta \left( S \right), \\ e_{7} & = 2\mu_{3} \left( {6\mu_{0}^{2} + 33\mu_{0} \mu_{1} - 3\mu_{0} \mu_{2} + 9\mu_{1}^{2} } \right)y\left( s \right) \\ & \quad + 2\mu_{2} \left( {27\mu_{1}^{2} - 27\mu_{1} \mu_{2} - 36\mu_{0} \mu_{2} + 27\mu_{0} \mu_{1} - 21\mu_{0} \mu_{3} } \right)\left( {y\left( S \right) - d_{3} sin \theta \left( S \right)} \right), \\ e_{8} & = 6\mu_{3} \left( {6\mu_{1}^{2} + 5\mu_{0} \mu_{1} + 4\mu_{0} \mu_{2} } \right)y\left( S \right) \\ & \quad + 6\mu_{2} \left( {6\mu_{0} \mu_{2} + 9\mu_{1} \mu_{2} + 9\mu_{1}^{2} + \mu_{0} \mu_{3} } \right)\left( {y\left( S \right) - d_{3} sin \theta \left( S \right)} \right), \\ e_{9} & = 12\mu_{3} \left( {6\mu_{1}^{2} + 4\mu_{0} \mu_{1} - \mu_{0} \mu_{3} + \mu_{0} \mu_{2} } \right)y\left( S \right) \\ & \quad + 6\mu_{2} \left( { 9\mu_{1}^{2} - 9\mu_{1} \mu_{2} - 9\mu_{0} \mu_{2} - 13\mu_{0} \mu_{3} - 9\mu_{1} \mu_{3} } \right)\left( {y\left( S \right) - d_{3} sin \theta \left( S \right)} \right), \\ e_{10} & = 6\mu_{3} \left( {6\mu_{1}^{2} + 9\mu_{1} \mu_{2} + \mu_{0} \mu_{3} + 4\mu_{0} \mu_{2} } \right)y\left( S \right) + 6\mu_{2} \left( {9\mu_{1} \mu_{2} + \mu_{0} \mu_{3} } \right)\left( {y\left( S \right) - d_{3} sin \theta \left( S \right)} \right), \\ e_{11} & = 18\mu_{3} \left( {\mu_{0} \mu_{2} - \mu_{0} \mu_{3} + 3\mu_{1} \mu_{2} - \mu_{1} \mu_{3} + 3\mu_{1}^{2} } \right)y\left( S \right) \\ & \quad + 6\mu_{2} \left( { - 6\mu_{0} \mu_{3} + 6\mu_{1} } \right)\left( {x\left( S \right) - d_{3} cos \theta \left( S \right)} \right), \\ e_{12} & = 6\mu_{3} \left( {3\mu_{2}^{2} + 9\mu_{1} \mu_{2} + \mu_{0} \mu_{3} + 2\mu_{1} \mu_{3} } \right)y\left( S \right) - 18\mu_{2}^{2} \mu_{3} \left( {y\left( S \right) - d_{3} sin \theta \left( S \right)} \right), \\ e_{13} & = - 6\mu_{3} \left( {\mu_{0} \mu_{3} - 9\mu_{1} \mu_{2} + 4\mu_{1} \mu_{3} + \mu_{2} \mu_{3} - 3\mu_{2}^{2} } \right)y\left( S \right) + ( - 18\mu_{3} \mu_{2}^{2} + 9\mu_{1} \mu_{2} \\ & \quad - \mu_{2} \mu_{3} )\left( {y\left( S \right) - d_{3} sin \theta \left( S \right)} \right), \\ e_{14} & = 6\mu_{3} \left( {3\mu_{2}^{2} + \mu_{2} \mu_{3} + 2\mu_{1} \mu_{3} } \right)y\left( S \right) - 6\mu_{3} \left( {3\mu_{2}^{2} + \mu_{2} \mu_{3} } \right)\left( {y\left( S \right) - d_{3} sin \theta \left( S \right)} \right), \\ e_{15} & = 6\mu_{2} \mu_{3}^{2} y\left( S \right) - 6\mu_{2} \mu_{3}^{2} \left( {y\left( S \right) - d_{3} sin \theta \left( S \right)} \right), \\ e_{16} & = - 6\mu_{3} \left( { - 3\mu_{2}^{2} + \mu_{2} \mu_{3} + \mu_{1} \mu_{3} } \right)y\left( S \right) - 6\mu_{3} \left( {3\mu_{2}^{2} - \mu_{2} \mu_{3} } \right)\left( {y\left( S \right) - d_{3} sin\theta \left( S \right)} \right). \\ \end{aligned} $$

Substituting the values from (28) and (29) into (3), the curvature of the RCTBC (1) is given by

30 $$ k_{p} \left( t \right) = \frac{E\left( t \right).I\left( t \right) - G\left( t \right).H\left( t \right)}{{F\left( t \right))\left\{ {\left( {E\left( t \right)} \right)^{2} + \left( {G\left( t \right)} \right)^{2} } \right\}^{{\frac{3}{2}}} }}. $$

Here, $$ E\left( t \right), $$$$ F\left( t \right),  G\left( t \right), $$$$ H\left( t \right) $$ and $$ I\left( t \right) $$ have been already defined. Maximum relative curvature error of $$ G^{2} $$-approximation while approximating GCS by the RCTBC (1) is calculated as,

31 $$ \delta \left( t \right) = max_{{t \in \left[ {0,\frac{\pi }{4}} \right]}} \overline{\delta \left( t \right)} , $$

whereas the relative curvature error of the developed $G^{2}$-approximation scheme of GCS is $\overline{\delta \left( t \right)} = \frac{{\left| {k_{p} \left( t \right) - k\left( S \right)} \right|}}{{max\left\{ {\left| {k_{p} \left( t \right)} \right|,\left| {k\left( S \right)} \right|} \right\}}}$. However, for sufficiently small values of the curvatures ($k_{p} \left( t \right),k\left( S \right)$), the relative curvature error $\overline{\delta \left( t \right)}$ becomes infinite. Therefore, the practical choice of $\overline{\delta \left( t \right)}$ is the following:

32 $$ \overline{\delta \left( t \right)} = \frac{{\left| {k_{p} \left( t \right) - k\left( s \right)} \right|}}{{max\left\{ {1,\left| {k_{p} \left( t \right)} \right|,\left| {k\left( s \right)} \right|} \right\}}} . $$

The curvature of the GCS $k\left( s \right)$ is given by

33 $$ k\left( s \right) = \frac{{(\tilde{k}_{1} - \tilde{k}_{0} + r\tilde{k}_{1} )s + \tilde{k}_{0} s}}{rs + S},\quad 0 \le s \le S,\quad  r > - 1. $$

Here $\tilde{k}_{0}$ and $\tilde{k}_{1}$ are the end curvatures of the GCS, $r$ is the shape factor, $S$ is the total arc length of the GCS and $s$ is the arc length parameter.

#### Determining bounds of free parameters

In order to determine the optimized values of the free parameters $$ d_{1} $$, $$ d_{3} , $$$$ \mu_{1} $$ and $$ \mu_{2} $$ by minimizing the relative curvature error (31) of the developed $$ G^{2} $$-approximation scheme, the bounds of these free parameters are determined in the rest of this section.

By simple computation it can be easily observed that the values of first order derivative of RCTBC (1) at the end points of its domain $\left[ {0,\frac{\pi }{4}} \right]$ are

34 $$ p'\left( 0 \right) = \frac{{3\mu_{1} \left( {p_{1} - p_{0} } \right)}}{{\mu_{0} }} \quad{\text{and}}\quad p'\left( {\frac{\pi }{4}} \right) = \frac{{6\mu_{2} \left( {p_{3} - p_{2} } \right)}}{{\mu_{3} }}. $$

Taking the norm on both sides and after some rearrangement, we have

35 $$ \| p_{1} - p_{0}\| = \frac{{\mu_{0} \|p'\left( 0 \right)\|}}{{3\mu_{1} }} \quad{\text{and}}\quad \|p_{3} - p_{2}\| = \frac{{\mu_{3} \left\|p'\left( {\frac{\pi }{4}} \right)\right\|}}{{6\mu_{2} }}. $$

Here $\| p_{1} - p_{0} \|$ is the distance between first two control points and $\|p_{3} - p_{2} \|$ is the distance between last two control points of (1). Since for a given GCS, $\| p'\left( 0 \right)\|$ and $\left\| p'\left( {\frac{\pi }{4}} \right) \right\|$ are fixed so for a reasonable distance between $p_{0} \left( {p_{3} } \right)$ and $p_{1} \left( {p_{2} } \right),$ the reasonable choice of the free parameters are $\mu_{1} \ge 1$ and $\mu_{2} \ge 1.$

The arc length of RCTBC (1) over the whole domain is given by

$$ S_{T} = \mathop \int \nolimits_{0}^{{\frac{\pi }{4}}} \|p'\left( t \right)\|dt. $$

The above integral cannot be solved analytically. Therefore numerical integration technique is needed to evaluate it. Here, the above definite integral is evaluated by Trapezoidal rule for $M = 2$ and $h = \frac{\pi }{16}$. *M* and $h$ represent the number of subintervals and length of each subinterval. The order of error of approximation of Trapezoidal rule is $\varvec{O}\left( {h^{2} } \right).$ The computed value of the arc length of the RCTBC (1) is

$$ \begin{aligned} S_{T} & \approx \frac{\pi }{16}\|p^{\prime}\left( 0 \right)\| + \frac{\pi }{8}\left\|p^{\prime}\left( {\frac{\pi }{8}} \right)\right\| + \frac{\pi }{16}\left\|p^{\prime}\left( {\frac{\pi }{4}} \right)\right\| \\ & > \frac{\pi }{16}\left\|p^{\prime}\left( 0 \right)\right\| + \frac{\pi }{16}\left\|p^{\prime}\left( {\frac{\pi }{4}} \right)\right\| \\ \end{aligned} $$

or

36 $$ \|p^{\prime}\left( 0 \right)\| < \frac{16}{\pi }S_{T}  \quad{\text{and}}\quad \left\|p^{\prime}\left( {\frac{\pi }{4}} \right)\right\| < \frac{16}{\pi }S_{T} $$

Substituting the values of $$ p_{i} , i = 0,1,2,3, $$ from (25) in (35), we have

37 $$ \|p^{\prime}\left( 0 \right) \|= \frac{{3\mu_{1} d_{1} }}{{\mu_{0} }} \quad{\text{and}}\quad \left\|p^{\prime}\left( {\frac{\pi }{4}} \right)\right\| = \frac{{6\mu_{2} d_{3} }}{{\mu_{3} }}. $$

Substituting the values of $\|p^{\prime}\left( 0 \right)\|$ and $\left\|p^{\prime}\left( {\frac{\pi }{4}} \right)\right\|$ from (37) into (36), we have

38 $$ \frac{{\mu_{1} d_{1} }}{{\mu_{0} }} < \frac{16}{3\pi }S_{T}  \quad{\text{and}}\quad \frac{{\mu_{2} d_{3} }}{{\mu_{3} }} < \frac{8}{3\pi }S_{T} . $$

Although the developed $$ G^{2} $$-approximation of GCS has four free parameters $$ d_{1} $$, $$ d_{3} $$$$ \mu_{1} $$ and $$ \mu_{2} $$ but its degrees of freedom are actually two (Yoshida and Saito 2007). Thus two out of four free parameters $$ d_{1} $$, $$ d_{3} $$, $$ \mu_{1} $$ and $$ \mu_{2} $$ can be chosen arbitrarily. It is because the scaling of control points and weights by same scale factor does the affect the shape of the curve (Yoshida and Saito 2007). Here for the $$ G^{2} $$-approximation of GCS by RCTBC (1), the weight functions $$ \mu_{1} $$ and $$ \mu_{2} $$ are fixed to $$ \mu_{1} = 1 $$ and $$ \mu_{2} = 1 $$ without loss of generality (Yoshida and Saito 2007). Thus there are only two free parameters $$ d_{1} $$ and $$ d_{3} $$ for the $$ G^{2} $$-approximation of GCS by rational cubic trigonometric Bézier curve (1). It follows from above discussion that for reasonable $$ G^{2} $$-approximation of GCS by RCTBC (1), the free parameters $$ d_{1} $$ and $$ d_{3} $$ should satisfy the following relation

39 $$ \frac{{d_{1} }}{{\mu_{0} }} < \frac{16}{3\pi }S_{T}  \quad{\text{and}}\quad \frac{{d_{3} }}{{\mu_{3} }} < \frac{8}{3\pi }S_{T} . $$

The above discussion is summarized as:

##### **Theorem 1**

*If the control points of the rational cubic trigonometric Bézier curve*$p\left( t \right)$, *defined in* (1), *are given by*

$$ \begin{aligned} p_{0} & = \left( {0,0} \right),\quad  p_{1} = \left( {d_{1} , 0} \right), \quad p_{2} = (x\left( S \right) - d_{3} cos \theta \left( S \right), y\left( S \right) - d_{3} sin \theta \left( S \right)), \,{\text{and}} \\  p_{3} & = \left( {x\left( S \right),y\left( S \right)} \right) ,\\ \end{aligned} $$

*and the two weight functions*$\mu_{0}$*and*$\mu_{3}$*are computed as*,

$$ \mu_{0} = \frac{{3\mu_{1}^{2} d_{1}^{2} \tilde{k}_{0} }}{{2\mu_{2} \left( {y\left( S \right) - d_{3} sin \theta \left( S \right)} \right)}},\quad \mu_{3} = \frac{{3\mu_{2}^{2} d_{3}^{2} \tilde{k}_{1} + y\left( S \right)cos \theta \left( S \right)}}{{2\mu_{1} sin \theta \left( S \right)\left( {x\left( S \right) - d_{1} } \right)}}, $$

*then the rational cubic trigonometric Bézier curve* (1) *gives the*$G^{2}$*-approximation of the GCS. Here the optimized values of the free parameters*$d_{1}$*and*$d_{3}$*are calculated from the following optimization problem-I.*

##### **Optimization problem-I**

$$ \begin{aligned} & {\text{Minimize}}\quad \delta \left( t \right) \\ & {\text{subject}}\,{\text{to}}\quad d_{1} \ge u,\quad d_{3} \ge u, \\ \end{aligned} $$

where

$$ \begin{aligned} \delta \left( t \right) & = max_{{t \in \left[ {0,\frac{\pi }{4}} \right]}} \overline{\delta \left( t \right)} , \\ \overline{\delta \left( t \right)} & = \frac{{\left| {k_{p} \left( t \right) - k\left( s \right)} \right|}}{{max\left\{ {1,\left| {k_{p} \left( t \right)} \right|,\left| {k\left( s \right)} \right|} \right\}}},\quad u = 2.2204 \times 10^{ - 16} . \\ \end{aligned} $$

For practical implementation of Theorem 1 and optimization problem-I, the optimized values of $$ d_{1} $$ and $$ d_{3} $$ are obtained from Optimization problem-I. These optimized values are substituted in Theorem 1 to obtain the corresponding values of $$ \mu_{0} $$ and $$ \mu_{3} . $$ If these computed values of $$ \mu_{0} , $$$$ \mu_{3} , $$$$ d_{1} $$ and $$ d_{3} $$ satisfy the inequalities in (39), then desired values are achieved. Otherwise the optimization problem-I is resolved with a different initial guess for $$ d_{1} $$ and $$ d_{3} $$.

### $G^{1}$-approximation of GCS

The $G^{1}$-approximation of GCS by rational cubic trigonometric Bézier curve (1) is carried out by the set of Eqs. (19) and (20). Hence, for the above mentioned $G^{1}$-approximation the control points $(p_{i} , i = 0,1,2,3)$ of the RCTBC (1) are the same as calculated in (25), “*G*^2^-approximation of GCS” section. Now all the weight functions ($\mu_{i} , i = 0,1,2,3)$ and $d_{1}$, $d_{3}$ are the free parameters (Yoshida and Saito 2007). Since the degree of freedom of RCTBC (1) is two less than its number of free parameters so two out of above six free parameters can be chosen arbitrarily (Yoshida and Saito 2007). For the ease of computation the weight functions $\mu_{0}$ and $\mu_{3}$ are fixed to $\mu_{0} = \mu_{3} = 1$. The for the $G^{1}$-approximation of GCS by rational cubic trigonometric Bézier curve (1) there are only four free parameters $d_{1}$, $d_{3}$, $\mu_{2}$ and $\mu_{3}$. The relation (38) yields the following bounds of these free parameters

40 $$ \mu_{1} d_{1} < \frac{16}{3\pi }S_{T}  \quad{\text{and}}\quad \mu_{2} d_{3} < \frac{8}{3\pi }S_{T} . $$

The optimized values of the free parameters $d_{1}$, $d_{3}$, $\mu_{2}$ and $\mu_{3}$ are obtained by minimizing the maximum value of relative curvature error of $G^{1}$-approximation scheme.

#### **Theorem 2**

*If the control points of the rational cubic trigonometric Bézier curve*$p\left( t \right)$, *defined in* (1), *are given by*

$$ \begin{aligned} p_{0} & = \left( {0,0} \right),\quad  p_{1} = \left( {d_{1} , 0} \right),\quad p_{2} = (x\left( S \right) - d_{3} cos \theta \left( S \right), y\left( S \right) - d_{3} sin \theta \left( S \right)), \,{\text{and}} \\ p_{3} & = \left( {x\left( S \right),y\left( S \right)} \right) ,\\ \end{aligned} $$

*then the rational cubic trigonometric Bézier curve* (1) *gives the*$G^{1}$-*approximation of the GCS. Here the optimized values of the free parameters*$d_{1} , d_{3} , \mu_{1}$*and*$\mu_{2}$*are calculated from the following optimization problem-II*.

#### **Optimization problem-II**

$$ \begin{aligned} & {\text{Minimize}}\quad \delta \left( t \right) \\ & {\text{subject}}\,{\text{to}}\quad d_{1} \ge u,\quad d_{3} \ge u, \\ & \mu_{1} \le \frac{16}{{3\pi d_{1} }}S_{T} - u,\quad \mu_{2} \le \frac{8}{{3\pi d_{3} }}S_{T} - u, \\ \end{aligned} $$

where

41 $$ \begin{aligned} \delta \left( t \right) & = max_{{t \in \left[ {0,\frac{\pi }{4}} \right]}} \overline{\delta \left( t \right)} ,\quad u = 2.2204 \times 10^{ - 16} \\ \overline{\delta \left( t \right)} & = \frac{{\left| {k_{p} \left( t \right) - k\left( s \right)} \right|}}{{max\left\{ {1, \left| {k_{p} \left( t \right)} \right|,\left| {k\left( s \right)} \right|} \right\}}}. \\ \end{aligned} $$

#### *Remark 1*

It is clear from the choice of $\overline{\delta \left( t \right)}$ of Theorems 1 and 2 that for very small values of $k_{p} \left( t \right)$ and $k\left( S \right)$, the free parameters are obtained by minimizing the maximum absolute curvature error of the proposed $G^{k}$-approximation schemes.

#### *Remark 2*

It can be observed from Theorems 1 and 2 that the control points, weight functions and curvature of RCTBC (2) for $G^{2}$ and $G^{1}$ approximation of the GCS are not dependent on the distance $d_{2}$.

#### *Remark 3*

For determining the bounds of free parameters in “*G*^2^-approximation of GCS” and “*G*^1^-approximation of GCS” sections, the length of GCS and its approximating RCTBC are taken approximately equal i.e. $S_{T} \approx S.$

#### *Remark 4*

In this research paper, the optimization problems I and II are solved by using the function program fminimax of the optimization toolbox of MATLAB 7 software. The fminimax is based on sequential quadratic programming technique (SQP) (Chong and Zak 2010). SQP is a state of art method of optimization. It is more efficient and accurate then the prevailing optimization techniques. In SQP, firstly, the Hessian matrix of Lagrangian function is updated to obtain a positive definite Hessian. This updating is carried out using quasi-Newton updating method preferably the BFGS algorithm. Secondly, updated Hessian is used to generate a quadratic programming sub-problem. The solution of quadratic programming sub-problem is used to determine search direction. Lastly, this search direction is used to obtain a new iterate by line search algorithm. The step length parameter of line search algorithm is determined by sufficient decrease in merit function. It is observed that the sequential quadratic programming technique is infeasible or fails for highly nonlinear and discontinuous objective functions. But the objective functions involved in Theorems 1 and 2 are neither highly nonlinear nor discontinuous, so feasible solutions of optimization problems I and II is possible.

## Re-parameterization of the GCS and the rational cubic trigonometric Bézier curve

The curvatures of GCS and the rational cubic trigonometric Bézier curve (1) can be easily compared if these curvatures have a common parameter. Since the curvature of the rational cubic trigonometric Bézier curve $k_{p} \left( t \right)$ has parameter $t \in \left[ {0,\frac{\pi }{4}} \right]$ while the curvature of the GCS $k\left( s \right)$ is expressed in terms of arc-length parameter $s$. Therefore in this research paper the curvatures are compared by matching corresponding points along the arc of the GCS and the Bézier curve (1). The developed algorithm is the modified version of the numerical algorithm presented in (Wang et al. 2003). The details are as follows:

### **Algorithm 1**

- **Step 1** Divide the interval $$ \left[ {0,\frac{\pi }{4}} \right] $$ into $$ m $$ equally spaced subintervals such that the length of each subinterval is $$ \frac{\pi }{4m} $$. Now, the partition of the interval $$ \left[ {0,\frac{\pi }{4}} \right] $$ is $$ 0 = t_{0} < t_{1} < t_{2} < \cdots < t_{m} = \frac{\pi }{4}, $$ where $$ t_{i + 1} = t_{i} + \frac{\pi }{4m}, $$$$ i = 0, 1, 2, \ldots , m - 1. $$
- **Step 2** Compute the arc length $\tilde{s}_{j}$ of RCTBC (1) over the subinterval $\left[ {t_{0} ,t_{j} } \right]$, $j = 1, 2,3, \ldots , m.$
- **Step 3** Divide the interval $\left[ {0,S} \right]$ into $p$ equally spaced subintervals where $S$ is the total arc length of the GCS. The partition of $\left[ {0,S} \right]$ is $0 = \bar{s}_{0} < \bar{s}_{1} < \bar{s}_{2} < \cdots < \bar{s}_{p} = S$, where $\bar{s}_{i + 1} = \bar{s}_{i} + \frac{S}{p}$, $i = 0, 1, 2, \ldots , p - 1$. Here $p$ and $m$ are positive integers with $p < m$ and $m$ is very large.
- **Step 4**
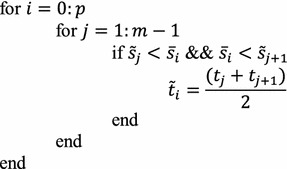
  Here $\tilde{t}_{i}$’s are the point on the RCTBC corresponding to the points $\bar{s}_{i}$ on the GCS.

### *Remark 5*

It is observed through numerical experiments that for acceptable approximation of GCS the suitable choice is $m > 700.$

All the above discussion is summarized in the form of following algorithm.

### **Algorithm 2**

- **Step 1.** Compute the curvature of rational cubic trigonometric curve (1) and GCS from Eqs. (30) and (33) respectively.
- **Step 2.** Compute the values of free parameters from optimization problems I and II for $G^{2}$ and $G^{1}$ approximation schemes respectively.
- **Step 3.** Given $x\left( S \right)$, $y\left( S \right)$ and $\theta \left( S \right)$, substitute the values of free parameters obtained in Step 2 to compute the control points corresponding to proposed $G^{2}$ and $G^{1}$ approximation schemes of GCS.
- **Step 4.** Put the values of control points obtained from Step 3 into (1) to obtain RCTB $$ G^{k} , k = 1,2, $$ approximation of GCS.

## Numerical examples

In this section, $G^{2}$ and $G^{1}$-approximation schemes developed in this research paper are tested for the special cases of GCS. For each data set of GCS, the maximum relative curvature errors for the proposed $G^{2}$ and $G^{1}$-approximation schemes are calculated and found less than the prevailing approximation schemes of GCS. The initial conditions for special cases of GCS are given in Table 2. The initial values of the free parameters for approximation are given in Table 3.

**Table 2:** Initial conditions for the special cases of GCS

Initial conditions	Circular arc	Cornu spiral	Logarithmic spiral	Non-inflecting GCS	Normalized GCS
*S*	1.5707	4.0	8.0	3.0	1
*r*	0.0	0.0	−0.75	0.4	0.1
*k*(0)	1.0	0.1	0.1	0.1	0.36
*k*(*S*)	1.0	0.5	0.4	0.5	2.7
*r*(0)	(0, 0)	(0, 0)	(0, 0)	(0, 0)	(0, 0)
*r*(*S*)	(1, 1)	(3.362, 1.680)	(6.199, 3.959)	(2.670, 1.076)	(0.7499, 0.4915)
*θ*(0)	0	0	0	0	0
*θ*(*S*)	1.5707	1.2	1.497	0.967	1.5672

**Table 3:** Initial guess of parameters and anticipated tolerance of approximation

Approximation schemes	Values of parameters/anticipated tolerance ($\varepsilon$)	Circular arc	Cornu spiral	Logarithmic spiral	Non-inflecting GCS	Normalized GCS
*G* ^2^-approximation	$d_{1}$	0.5	1.515	5	1.1	2
	*d*_3_	0.5	1.426	1.7	1.1	2
	$\varepsilon$	3.59 × 10^−2^	2.78 × 10^−2^	6.42 × 10^−2^	1.10 × 10^−2^	0.05
*G* ^1^-approximation	$d_{1}$	0.457	1.45	2.28	1.089	2
	*d*_3_	0.688	1.40	3.19	0.899	2
	*μ*_1_	0.876	0.67	0.88	1.55	0.8
	*μ*_2_	0.812	0.899	0.787	1.32	0.8
	$\varepsilon$	3.59 × 10^−2^	2.78 × 10^−2^	6.42 × 10^−2^	1.10 × 10^−2^	0.05

### *Example 1*

**Fig. 2:**
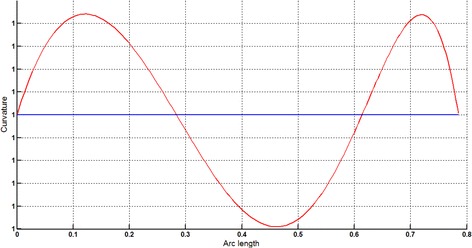
Curvature plot: circular arc versus Bézier curve

**Fig. 3:**
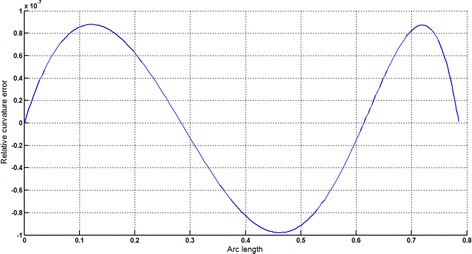
Relative curvature error

**Fig. 4:**
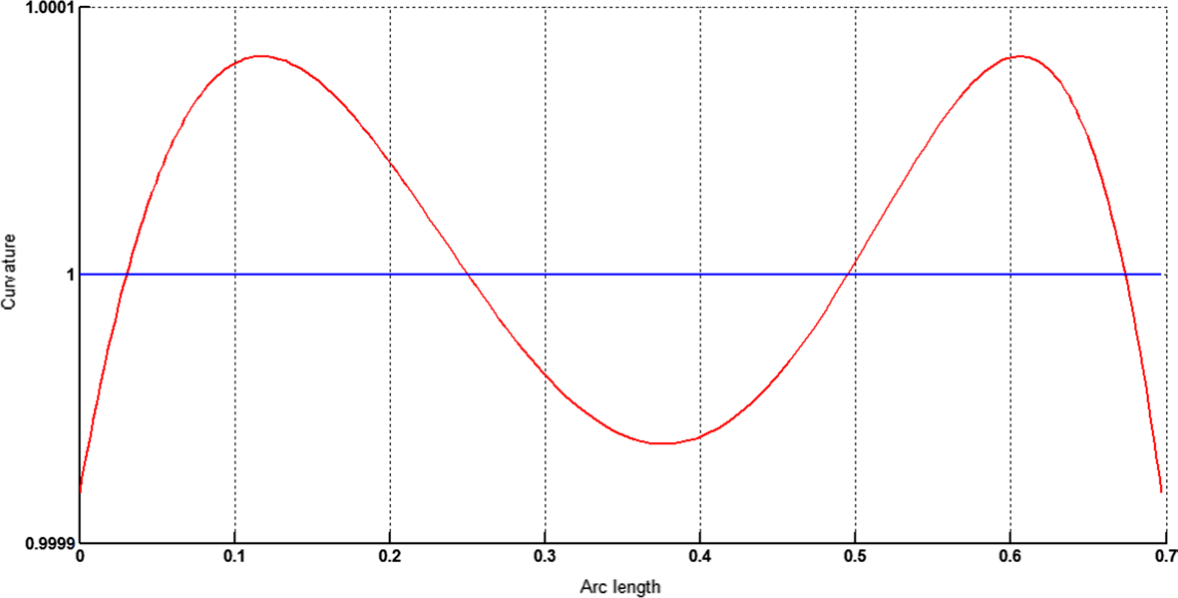
Curvature plot: circular arc versus Bézier curve

**Fig. 5:**
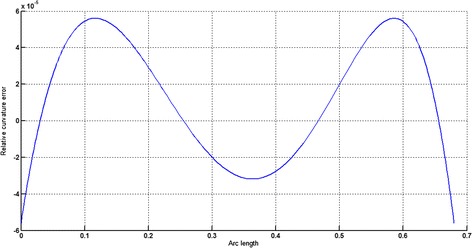
Relative curvature error

The circular arc given in Table 2 is approximated by Theorems 1 and 2 respectively. The curvature plots of circular arc and its $G^{2}$-approximation by RCTBC are given in Fig. 2. Similarly, curvature plots of circular arc and its $G^{1}$-approximation by RCTBC are given in Fig. 4. It is clear from Figs. 2 and 4 that the curvatures of the circular arc and its RCTB approximations are nearly identical. The relative curvature errors plots of the $G^{2}$ and $G^{1}$ approximations of the concerned circular arc are given in Figs. 3 and 5 respectively.

### *Example 2*

**Fig. 6:**
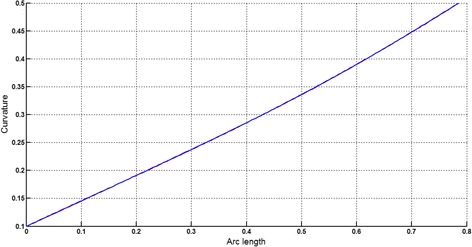
Curvature plot: Cornu spiral versus Bézier curve

**Fig. 7:**
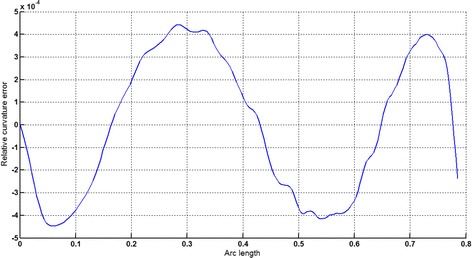
Relative curvature error

**Fig. 8:**
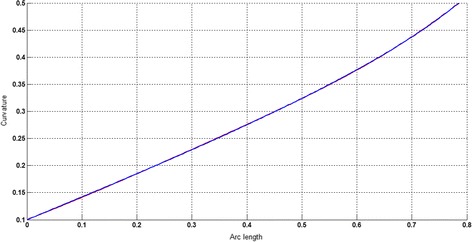
Curvature plot: Cornu spiral versus Bézier curve

**Fig. 9:**
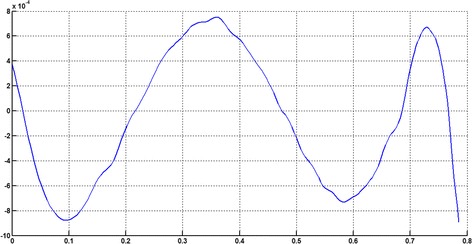
Relative curvature error

The Cornu spiral arc given in Table 2 is approximated by Theorems 1 and 2 respectively. The curvature plots of Cornu spiral and its $G^{2}$-approximation by RCTBC are given in Fig. 6. Similarly, curvature plots of Cornu spiral and its $G^{1}$-approximation by RCTBC are given in Fig. 8. It is clear from Figs. 6 and 8 that the curvatures of the Cornu spirals and its RCTBC approximations are overlapping. The relative curvature errors plots of the $G^{2}$ and $G^{1}$ approximations of the concerned arc of Cornu spiral are given in Figs. 7 and 9 respectively.

### *Example 3*

**Fig. 10:**
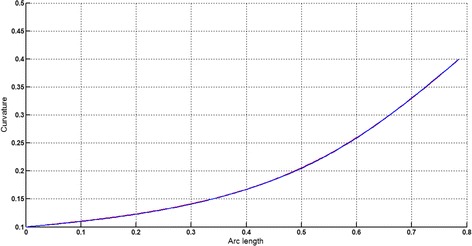
Curvature plot: logarithmic spiral versus Bézier curve

**Fig. 11:**
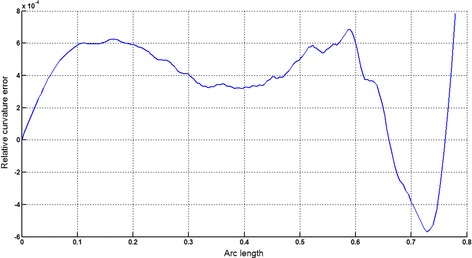
Relative curvature error

**Fig. 12:**
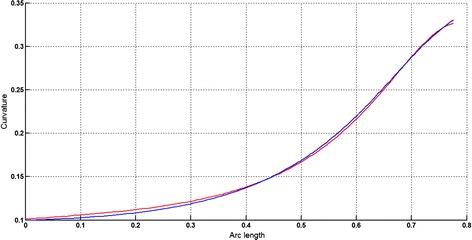
Curvature plot: logarithmic spiral versus Bézier curve

**Fig. 13:**
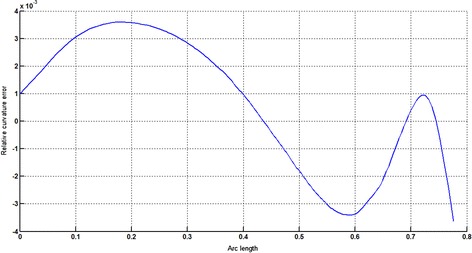
Relative curvature error

The logarithmic spiral given in Table 2 is approximated by Theorems 1 and 2 respectively. The curvature plots of logarithmic spiral and its $G^{2}$-approximation by RCTBC are given in Fig. 10. Similarly, curvature plots of logarithmic spiral and its $G^{1}$-approximation by RCTBC are given in Fig. 12. It is clear from Figs. 10 and 12 that the curvatures of the logarithmic spiral and its RCTB approximations are overlapping. The relative curvature errors plots of the $G^{2}$ and $G^{1}$ approximations of the concerned logarithmic spiral arc are given in Figs. 11 and 13 respectively.

### *Example 4*

**Fig. 14:**
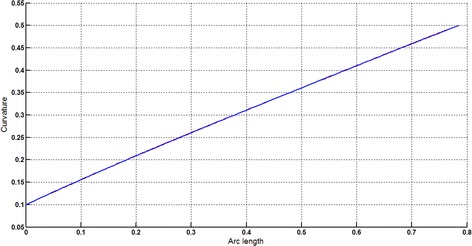
Curvature plot: non-inflecting GCS versus Bézier curve

**Fig. 15:**
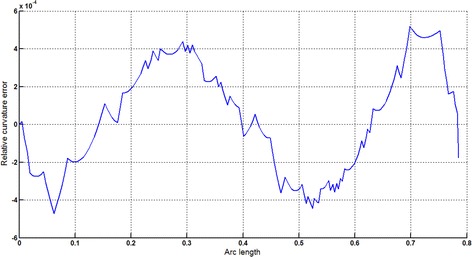
Relative curvature error

**Fig. 16:**
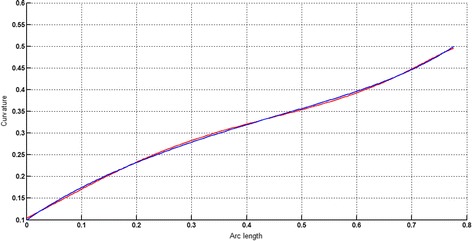
Curvature plot: non-inflecting GCS versus Bézier curve

**Fig. 17:**
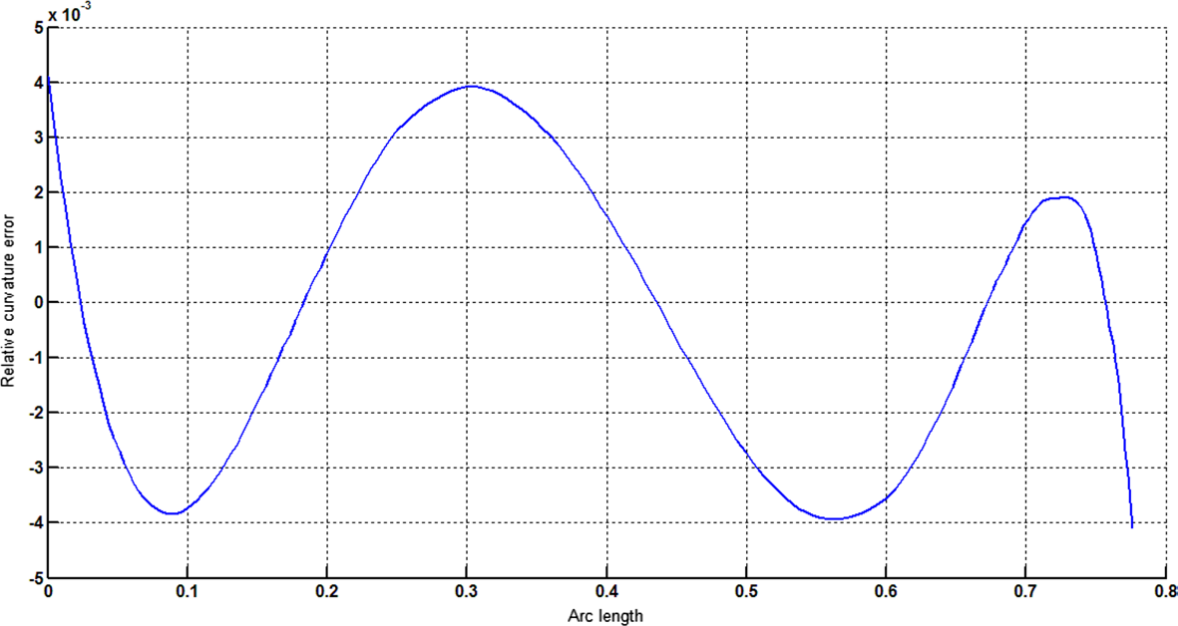
Relative curvature error

The non-inflecting GCS given in Table 2 is approximated by Theorems 1 and 2 respectively. The curvature plots of non-inflecting GCS and its $G^{2}$-approximation by RCTBC are given in Fig. 14. Similarly, curvature plots of non-inflecting GCS and its $G^{1}$-approximation by RCTBC are given in Fig. 16. It is clear from Figs. 14 and 16 that the curvatures of the non-inflecting GCS and its RCTB approximations are overlapping. The relative curvature errors plots of the $G^{2}$ and $G^{1}$ approximations of the concerned non-inflecting GCS are given in Figs. 15 and 17 respectively.

### *Example 5*

**Fig. 18:**
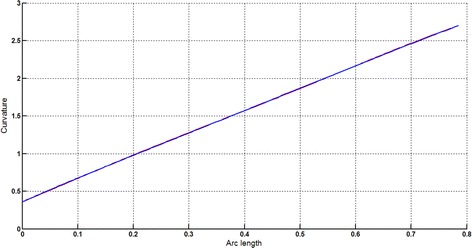
Curvature plot: normalized GCS versus Bézier curve

**Fig. 19:**
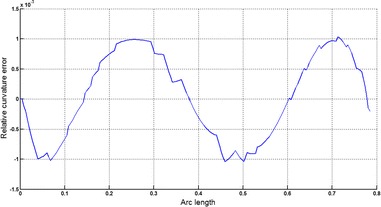
Relative curvature error

**Fig. 20:**
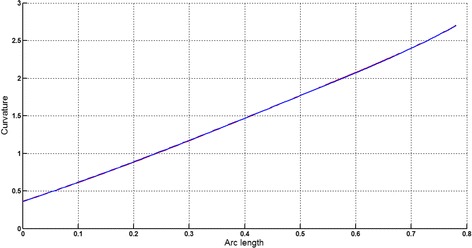
Curvature plot: normalized GCS versus Bézier curve

**Fig. 21:**
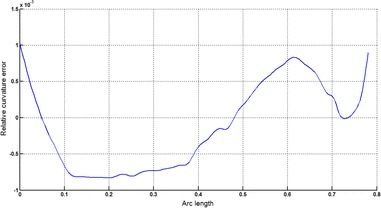
Relative curvature error

The normalized GCS of Table 2 is first approximated by the Theorem 1. Figure 18 expresses the curvature plots of the normalized GCS and the RCTBC approximating it. It is clear from the Fig. 18 that curvatures of actual and approximating curves are nearly identical. The relative curvature error plot of the $G^{2}$-approximation of the GCS is given in Fig. 19. The same normalized GCS of Table 2 is approximated by the $G^{1}$-approximation scheme presented in Theorem 2. For the $G^{1}$-approximation of the normalized GCS, the curvature plots of the actual and the approximating RCTBC are overlapping each other in Fig. 20. The relative curvature error plot of the $G^{1}$-approximation of inflecting GCS is given in the Fig. 21.

The circular arc, Cornu spiral, logarithmic spiral and non-inflecting GCS of Table 2 were also approximated by the $$ G^{2} $$-approximation scheme (Cripps et al. 2010). The relative curvature error of approximation of the $$ G^{2} $$-approximation scheme (Cripps et al. 2010) was $$ 7.75 \times 10^{ - 5} , $$$$ 1.25 \times 10^{ - 3} , $$$$ 8.80 \times 10^{ - 3} $$ and $$ 10^{ - 3} $$ for circular arc, Cornu spiral, logarithmic spiral and non-inflecting GCS respectively. A comparison of the above values of relative curvature error with Table 1 shows that $$ G^{k} $$-approximation schemes proposed in this research paper perform better than Cripps et al. (2010).

The optimum values of the free parameters of the developed $G^{2}$ and $G^{1}$ approximation schemes for the Examples 1–5 are summarized in Table 4. It is clear from Table 1 that the developed $G^{2}$ and $G^{1}$ approximation schemes provide favourable results.

**Table 4:** Optimum values of free parameters

Approximation schemes	Values of parameters/anticipated tolerance ($\varepsilon$)	Circular arc	Cornu spiral	Logarithmic spiral	Non-inflecting GCS	Normalized GCS
*G* ^2^-approximation	$d_{1}$	0.5838	1.5259	4.4089	1.1203	1.9710
	*d*_3_	0.5877	1.4252	1.8697	1.0948	2.0146
*G* ^1^-approximation	$d_{1}$	1.7	1.4	1.09	1.4	2.1202
	*d*_3_	1.7	1.4	1.09	1.4	1.9523
	*μ*_1_	0.7770	0.999	0.555	1.111	0.8183
	*μ*_2_	0.7770	0.999	0.555	1.111	0.6526

### *Remark 6*

The developed $G^{2}$-approximation scheme ensures point, tangent and curvature continuity whereas the developed $G^{1}$-approximation scheme preserves point and tangent continuity. The $G^{2}$-approximation scheme invokes more appreciable curvature plots than the developed $G^{1}$-approximation scheme. The degrees of freedom of $G^{2}$ and $G^{1}$ approximation schemes are two and four respectively. Therefore, the CPU time consumption of $G^{2}$-approximation scheme is less than $G^{1}$-approximation scheme, see the Table 1. It follows that the developed $G^{2}$-approximation scheme is better than the $G^{1}$-approximation scheme.

## Conclusion

In this research paper, $G^{2}$ and $G^{1}$ approximation schemes of GCS are developed using RCTBC (1). The choice of these approximation schemes serves the purpose of favourable approximations with minimized errors, see Table 1. The CPU time consumed by the developed trigonometric approximation schemes is acceptable. The observations given in Table 1 convey that the $G^{2}$-approximation scheme works better than the developed $G^{1}$-approximation scheme.
